# Drug‐Coated Balloon (Optilume^®^) Shows a Low Reintervention Rate in Patients With Bulbar Urethral Strictures: Real‐World Data From Two German Centers

**DOI:** 10.1155/aiu/8510976

**Published:** 2026-03-22

**Authors:** Juan Jose Menendez-Suarez, Georgi Tosev, Hendrik Borgmann, Johannes Salem, Timur Kuru

**Affiliations:** ^1^ Department of Urology, CUROS Urology Center, Cologne, Germany; ^2^ Department of Urology, Praxis Urologie Mannheim, Mannheim, Germany; ^3^ Department of Urology, University Hospital Heidelberg, Heidelberg, Germany, heidelberg-university-hospital.com; ^4^ Department of Urology, Faculty of Health Sciences Brandenburg, Brandenburg Medical School Theodor Fontane, Brandenburg, Germany

**Keywords:** bulbar urethral stricture, drug-coated balloon, mid-term outcomes, minimally invasive treatment, urethral stenosis, urethroplasty alternative

## Abstract

**Background:**

Bulbar urethral strictures are a common cause of lower urinary tract symptoms (LUTSs) in men and frequently recur after standard endoscopic treatments. There is growing interest in identifying less invasive techniques that provide durable outcomes compared to conventional endoscopic methods. Optilume has shown favorable outcomes in prospective trials, but real‐world evidence, especially including treatment‐naïve patients, remains limited.

**Methods:**

We performed a retrospective study in two German urology practices, including 40 men with symptomatic bulbar urethral strictures. Symptoms were assessed using the International Prostate Symptom Score (IPSS) and IPSS‐derived quality‐of‐life (QoL) score at baseline and at follow‐up. The primary endpoints were changes in IPSS and QoL; secondary endpoints included reintervention and safety. Nonparametric paired testing (Wilcoxon) and correlation analysis (Pearson) were applied.

**Results:**

Median follow‐up was 29.5 months. IPSS improved from a median of 19.5 (2–35) to 6.0 (0–26), and QoL improved from 5.0 (0–6) to 1.0 (0–5) (*p* < 0.000001). The median absolute change was ΔIPSS −10.5 overall; ΔIPSS was −16.5 in treatment‐naïve patients and −9.5 in previously treated patients. Median ΔQoL was −4.0 overall (−3.5 treatment‐naïve; −4.0 previously treated). One patient (2.5%) was scheduled for reintervention due to subjective dissatisfaction despite IPSS improvement. Treatment‐naïve patients showed a trend toward better outcomes.

**Conclusions:**

Optilume demonstrated excellent mid‐term outcomes (median follow‐up: 29.5 months) in the treatment of bulbar urethral strictures, with significant improvements in symptom burden and QoL and a low reintervention rate (2.5%) in a real‐world two‐center cohort with a nonsignificant trend toward greater symptomatic improvement in treatment‐naïve individuals. These findings support Optilume as a viable minimally invasive option. Larger prospective studies incorporating stricture‐specific patient‐reported outcome measures and comparative designs are needed to confirm long‐term effectiveness, cost considerations, and optimal patient selection.

## 1. Introduction

The male urethra is anatomically divided into two segments: anterior, which contains spongy tissue, and posterior, where it is absent [1]. Approximately 1 in 100 males will develop a urethral stricture [2], with the bulbar region being the most affected. The remodeling and spongiofibrosis that occur following tissue damage make repairing strictures particularly challenging.

In contemporary practice, urethral strictures are most commonly caused by iatrogenic injury, idiopathic fibrosis, or trauma and are associated with a substantial impact on quality of life (QoL) and healthcare utilization due to frequent recurrences [1].

Standard endoscopic treatments such as urethral dilation and direct‐vision internal urethrotomy (DVIU) remain widely used as first‐line options for bulbar urethral strictures. While open urethroplasty offers superior long‐term success, its invasiveness and perioperative morbidity limit its acceptance among many patients [3–6]. In contrast, multiple studies have demonstrated high recurrence rates and limited long‐term durability of endoscopic treatments, particularly in recurrent or longer strictures [7–11]. These limitations have driven growing interest in less invasive treatment alternatives.

Steenkamp et al. reported that both dilation and DVIU provide similar outcomes in terms of success, but their efficacy diminishes with longer strictures, declining from approximately 60% success in short strictures to below 40% in longer lesions [7]. Most studies report a median time to recurrence of less than 12 months [7–11].

Paclitaxel‐coated balloon technology enables local delivery of an antiproliferative agent to the urethral wall, aiming to reduce scar formation and stricture recurrence after dilation while minimizing systemic exposure. Previous pharmacokinetic studies have demonstrated minimal systemic absorption, supporting the safety of this approach [1, 2, 12].

Drug‐coated balloon dilation represents a minimally invasive treatment option for patients with bulbar urethral strictures, particularly for those who decline urethroplasty or are not suitable candidates for more invasive reconstructive surgery.

Recent prospective trials have demonstrated the efficacy of paclitaxel‐coated balloon dilation for recurrent bulbar urethral strictures. The ROBUST I study reported sustained symptomatic improvement and low reintervention rates at 5 year follow‐up, while ROBUST III confirmed the superiority of drug‐coated balloon dilation compared to standard endoscopic treatment at 3 years [13, 14]. However, these trials were conducted in highly selected patient populations under controlled study conditions. Consequently, real‐world data reflecting routine clinical practice, broader patient characteristics, and everyday clinical decision‐making remain limited [15].

In particular, outcomes in broader patient populations, including treatment‐naïve individuals treated outside of trial protocols, are insufficiently characterized. The present study aims to address this gap by reporting real‐world outcomes of Optilume treatment from two German urology centers.

## 2. Methods

We performed a single‐arm, retrospective, two‐center, nonrandomized, open‐label investigation at two German urological practices to evaluate the effects of the DCB on lower urinary tract symptoms (LUTSs) and associated morbidity in patients with bulbar urethral strictures. Symptom burden was assessed using the International Prostate Symptom Score (IPSS) and QoL metrics, evaluated preoperatively and at follow‐up. Follow‐up duration varied between patients, reflecting routine clinical practice.

All patients completed and returned their postoperative questionnaires on 1 September 2024, which represents the uniform end of follow‐up for the entire cohort. Follow‐up duration (time from intervention to 1 September 2024) therefore varied between patients. Patient records from 22 January 2021 to 1 September 2024 were reviewed. Data cleaning and statistical analysis were performed in January 2025.

Men aged 18 years or older with at least one symptomatic bulbar urethral stricture, confirmed via cystoscopy or retrograde urethrography, were included. The dataset reflected a real‐world population, including individuals with prior pelvic radiation, urinary stones, or previous endourological interventions such as TURP, TURB, catheterization, HoLEP, or even prostatectomy. The baseline characteristics of the study population are summarized in Supporting Table S1.

For subgroup analyses, patients were stratified into treatment‐naïve individuals (no prior endoscopic or surgical intervention for urethral stricture disease) and previously treated patients who had undergone one or more prior interventions. Baseline characteristics for these subgroups are summarized in Table 1.

**TABLE 1:** Baseline characteristics stratified by prior treatment status.

Variable	Overall cohort (*n* = 40)	Treatment‐naïve (*n* = 16)	Previously treated (*n* = 24)
Age (years), median (range)	66.2 (24.3–90.2)	62.0 (24.3–80.7)	66.5 (26.8–90.2)
Stricture length (cm), median (range)	1.5 (0.5–4.0)	1.25 (0.5–3.0)	1.75 (0.5–4.0)
Follow‐up duration (months), median (range)	29.5 (9–44)	24 (9–44)	30 (11–44)
Number of prior interventions, median (range)	1 (0–8)	0 (0–0)	1 (1–8)
Etiology (*n* [%])			
─Iatrogenic	24 (60.0%)	8 (50.0%)	16 (66.7%)
─Idiopathic	15 (37.5%)	8 (50.0%)	7 (29.2%)
─Traumatic	1 (2.5%)	0 (0%)	1 (4.2%)

*Note:* Detailed iatrogenic subcategories (e.g., postprostatectomy or postirradiation) were not systematically recorded in this retrospective dataset and therefore could not be reported separately; most iatrogenic strictures followed prior transurethral procedures or urinary tract instrumentation.

The primary endpoints were changes in IPSS and QoL following treatment. Secondary outcomes included (1) reintervention‐free survival and (2) perioperative safety outcomes (intraoperative and postoperative adverse events). All patients provided written informed consent prior to enrollment, and the study was approved by the institutional ethics committee (approval number: F‐2023‐048). Statistical analysis was carried out using IBM SPSS Statistics Version 29.0 (2022), and a two‐tailed *p* value < 0.05 was considered statistically significant.

All procedures were conducted by experienced urologists (> 500 endourological procedures performed) using a standardized protocol. Antibiotic prophylaxis was initiated 48 h before and maintained for 72 h after the intervention. A baseline retrograde urethrogram was performed to evaluate stricture length and morphology. A rigid cystoscope (20 Fr, 0° lens, Karl Storz or Richard Wolf) was introduced, and a 150‐cm hydrophilic guidewire (Radifocus Terumo) was advanced carefully through the stenotic segment.

**FIGURE 1:**
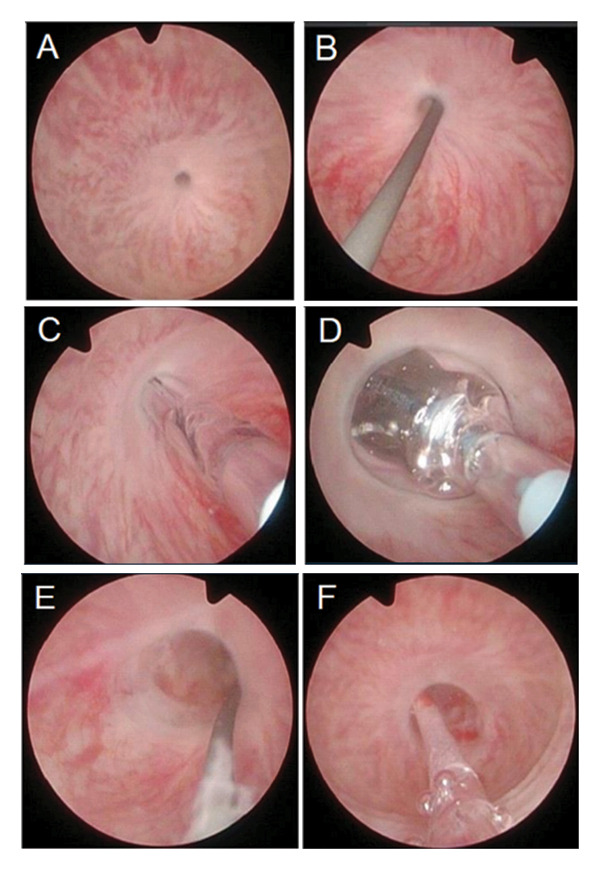
Endoscopic sequence of Optilume balloon urethroplasty for recurrent urethral stricture. (A) Identification of the stricture. (B) Advancement of the guidewire through the narrowed lumen. (C) Passage of the predilation balloon over the wire. (D) Inflation of the uncoated predilation balloon. (E) Achievement of > 50% luminal enlargement. (F) Introduction of the drug‐coated Optilume balloon into the dilated segment for final therapeutic dilation.

**FIGURE 2:**
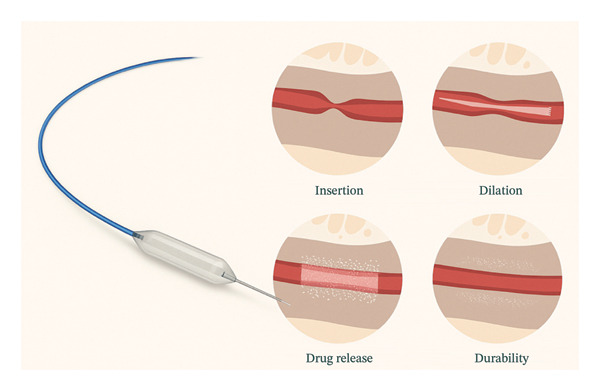
Left: the Optilume paclitaxel‐coated balloon device featuring a specialized balloon catheter with a blue guidewire and drug‐coated surface. Right: sequential illustration of therapeutic phases. (1) Insertion: initial placement of the deflated balloon in the strictured area. (2) Dilation: mechanical expansion of the strictured segment. (3) Drug release: transfer of paclitaxel coating into the surrounding tissue. (4) Durability: maintenance of luminal patency following treatment, with a sustained drug effect in tissue. Images adapted from G. Tosev, I. Damgov, and F. Kuehhas et al., off‐label use of the Optilume drug‐coated balloon in the treatment of bladder neck stenosis and vesicourethral anastomosis stenosis, Eur Urol Open Sci (2025).

A 24‐Fr uncoated balloon (UroMax) was used initially to dilate the urethra by approximately 50%. If no significant mucosal damage or bleeding was noted during this predilation, the DCB (5‐cm length and 30‐Fr diameter) was then deployed and inflated to 10 atmospheres for at least 7 min. In select cases with longer or concomitant strictures, the same technique was applied to each segment, using a new DCB for every dilation. A 14‐Fr Foley catheter was inserted for 48 h postoperatively to promote mucosal healing and adequate urinary drainage. As illustrated in Figures 1 and 2, the procedure entails guidewire placement, sequential predilation, and final drug‐coated balloon dilation to restore urethral patency. As per current guideline philosophy and in line with EAU recommendations, no routine anatomical follow‐up was performed in asymptomatic patients; instead, follow‐up focused on patient‐reported symptom relief, absence of retreatment, and overall satisfaction rather than routine anatomical assessment.

Patients who received Optilume as a first‐line treatment did so after thorough consultation, having declined open urethroplasty due to its greater invasiveness, the need for general anesthesia, longer hospitalization, and extended catheterization periods. In all such cases, Optilume was selected as a less invasive alternative aligned with patient preferences and informed consent. All patients completed a clinical visit with the IPSS questionnaire prior to treatment and again in January 2025.

## 3. Results

### 3.1. Study Population

We included 40 patients with bulbar urethral strictures, with a mean age of 59.95 ± 17.79 years (range: 24.3–90.2). Most patients (60%, *n* = 24) had iatrogenic strictures, followed by 37.5% (*n* = 15) with idiopathic strictures and 2.5% (*n* = 1) with a traumatic stricture. The mean preoperative IPSS was 20.30 ± 8.08, with 55% of patients (*n* = 22) reporting severe LUTS (IPSS > 19). The mean preoperative QoL score was 4.40 ± 1.19, indicating that many patients rated their QoL as “unhappy” or “terrible” (QoL 5–6). A total of 40% (*n* = 16) had no prior interventions, 35% (*n* = 14) had undergone one previous treatment, and 22.5% (*n* = 9) had undergone ≥ 2 procedures. The mean follow‐up duration was 27.65 ± 11.92 months (range: 9–44). Baseline demographic and clinical characteristics are summarized in Supporting Table S1.

We performed a statistical analysis to evaluate the clinical efficacy of the intervention. The primary outcomes included changes in IPSS and QoL. Secondary analyses explored the influence of prior interventions and stricture length on treatment outcomes.

### 3.2. Symptom Improvement

The evaluation of treatment outcomes demonstrated a statistically significant and clinically relevant reduction in both urinary symptoms and disease‐related QoL impairment (Table 2). The mean percentage improvement in IPSS was 56.3% (range: −50.0%–100.0%), with one patient (2.5%) achieving complete symptom resolution (IPSS 35–0).

**TABLE 2:** Summary of results for IPSS, IPSS QoL, % of improvement, and reintervention at baseline and follow‐up, with the Wilcoxon test.

Parameter	Baseline	Posttreatment at follow‐up (Jan 2025)	Wilcoxon test
IPSS			
Patients	40	40	
Median (range)	19.5 (2–35)	6.0 (0–26)	
% Improvement	—	56.3%	*p* < 0.000001
IPSS QoL			
Patients	40	40	
Median (range)	5.0 (0–6)	1.0 (0–5)	
% Improvement	—	57.5%	*p* < 0.000001
Reintervention	—	1/40 (2.5%)	—
Follow‐up, mo	—	Median 29.5 (9–44)	—

Only one patient (2.5%) showed a negative change in IPSS after treatment. However, this case is not considered clinically meaningful, as the baseline IPSS was already very low (2 points), and the posttreatment score increased by just one point (to 3). The −50% change reflects the mathematical effect of percentage calculation on small baseline values rather than a substantial clinical deterioration. Importantly, this patient reported a baseline QoL score of 6 (“terrible”), which improved significantly to 1 after treatment, indicating a clear subjective benefit.

The Wilcoxon signed‐rank test confirmed that the reduction in IPSS was statistically significant (*p* < 0.000001).

In terms of QoL, the mean percentage improvement was 57.5% (range: −200.0%–100.0%). Most patients (31 out of 40, 77.5%) experienced a meaningful improvement in QoL, while 6 patients (15.0%) showed minimal or no change, and 2 patients (5.0%) experienced worsening. Nonetheless, the overall improvement was statistically significant according to Wilcoxon testing (*p* < 0.000001).

In terms of absolute symptom improvement, the median change in IPSS (ΔIPSS) from baseline to follow‐up was −10.5 points in the overall cohort. When stratified by prior treatment status, median ΔIPSS was −16.5 points in treatment‐naïve patients and −9.5 points in previously treated patients.

Similarly, the median absolute change in IPSS‐related QoL score was −4.0 points overall, with median ΔQoL of −3.5 points in treatment‐naïve patients and −4.0 points in previously treated patients.

### 3.3. Subgroup Analysis by Number of Prior Interventions

Patients were stratified according to whether they had undergone any previous endoscopic treatment for urethral stricture. Among patients without prior interventions (*n* = 16), the median IPSS improvement was 76.0% (range: 0.0%–100.0%), and the QoL score improved by a median of 77.5%. In this subgroup, all patients experienced either symptom improvement or stability in both IPSS and QoL domains.

Among patients who had received at least one previous treatment (*n* = 24), the median IPSS improvement was slightly lower at 62.0% (range: −50.0%–96.3%), and the median QoL improvement was 80.0%. A small number of these patients showed limited or no improvement, and one patient experienced a mild increase in IPSS, as discussed previously. Although there was a trend toward better outcomes in treatment‐naïve patients, the difference did not reach statistical significance, possibly due to the limited sample size. Nevertheless, these findings suggest that Optilume is effective regardless of prior treatment history, with slightly more favorable responses observed in previously untreated individuals.

### 3.4. Impact of Stricture Length

To determine whether the length of the stricture influenced clinical outcomes, Pearson correlation analysis was conducted. No significant correlation was found between stricture length and improvement in IPSS (*r* = −0.16, *p* = 0.33) or QoL (*r* = −0.11, *p* = 0.52) (see Supporting Figure S1). This indicates that within the studied range, the effectiveness of the treatment was independent of stricture length.

### 3.5. Follow‐Up and Reintervention Rates

All 40 patients were followed for a median duration of 29.5 months (range: 9–44 months). During this period, one patient was programmed for reintervention, corresponding to a 2.5% reintervention or failure rate. The reintervention had been scheduled but had not yet taken place by the end of the follow‐up period; therefore, the exact time interval from the index procedure cannot be reported. In this case, the patient exhibited a clinical improvement in urinary symptoms, with a reduction in IPSS from 22 to 17, but reported a subjective worsening in QoL, with QoL increasing from 1 to 3, which motivated the decision for reintervention.

In one patient, recurrent symptoms were observed during follow‐up, and reintervention was discussed. However, after shared decision‐making, the patient explicitly declined further endoscopic or surgical treatment. Consequently, 39 patients (97.5%) did not require any additional procedures. No intraoperative or postoperative complications were observed in this cohort.

## 4. Discussion

Standard endoscopic treatments such as urethral dilation and DVIU are widely used as first‐line options for bulbar urethral strictures, although long‐term durability is limited. In our real‐world cohort, treatment with a paclitaxel‐coated balloon resulted in significant improvements in IPSS and QoL, with a low reintervention rate of 2.5% over a median follow‐up of 29.5 months. These findings are consistent with mid‐term outcomes reported in the ROBUST I and III trials [13, 14].

While dilation and DVIU are less invasive than urethroplasty, they are associated with high recurrence rates and short time to recurrence in most series [13, 16, 17]. In a large cohort study, Harraz et al. reported a patency rate (absence of reintervention) of 58% at a median follow‐up of 29 months [18]. In contrast, only one patient (2.5%) in our cohort required retreatment over a comparable follow‐up period.

The ROBUST III trial demonstrated superior patency with Optilume compared to standard dilation or DVIU in a randomized setting, but was conducted predominantly in patients with prior failed endoscopic treatments. In our cohort, some patients received Optilume as a first‐line therapy and tended to show slightly better outcomes, although this was not statistically significant. This trend may reflect the benefit of avoiding repeated instrumentation, which is known to increase fibrosis and stricture complexity [17, 19]. Importantly, prior intervention history did not significantly affect outcomes, supporting the utility of this approach even in previously treated patients.

Stricture length did not correlate significantly with treatment response, suggesting that outcomes were not limited to shorter lesions.

In terms of safety, no serious adverse events were observed in our series. This mirrors findings from the ROBUST trials [13, 14] and systematic reviews [20].

Importantly, one patient in our series showed a mild worsening in IPSS posttreatment while reporting an improved QoL. This highlights the multifactorial nature and lack of symptom specificity of the IPSS, which encompasses a variety of urinary symptoms that may not all have the same impact on daily life. For example, resolution of bothersome nocturia combined with increased daytime frequency may result in a higher overall IPSS score, yet still be perceived by the patient as an improvement, leading to a more satisfying QoL. However, the IPSS is not a stricture‐specific instrument and may insufficiently capture stricture‐related symptoms and should be interpreted as patient‐centered measures rather than definitive surrogates of urethral patency. Recently developed and validated stricture‐specific PROMs, such as the urethral stricture surgery PROM (USS‐PROM) and urethral stricture symptoms and impact measure (USSIM), may provide a more nuanced assessment and should be incorporated in future studies.

The definition of treatment success in bulbar urethral stricture disease remains nonstandardized. The EAU highlights that commonly used endpoints such as urethral patency, whether assessed radiologically, endoscopically, or by symptom resolution, do not always correlate with the patient’s perceived outcome [21, 22] (EAU Guidelines 2025; LE 3, SR C). Functional issues such as postvoid dribbling, ejaculatory symptoms, or discomfort may persist despite an anatomically open lumen. For this reason, the EAU recommends reporting patency or recurrence rates rather than using the ambiguous term “success.” Importantly, asymptomatic recurrences frequently do not require intervention. While luminal diameters below 10 French are generally associated with impaired flow [23], studies have shown that many strictures over 16 Fr remain stable and asymptomatic [23]. For example, in a prospective observational study by Purohit et al., 42 subclinical strictures > 16 Fr were followed over 23 months; only 12% showed progression, and none became symptomatic or required treatment. Likewise, Erickson et al. demonstrated that even when anatomic recurrence (< 16 Fr) was detected posturethroplasty, 35% of patients were asymptomatic [24].

Furthermore, routine use of invasive follow‐up tools such as cystoscopy or retrograde urethrography may provide limited clinical benefit while carrying risks including infection, bleeding, or iatrogenic trauma. A follow‐up strategy centered on functional outcomes and patient satisfaction is therefore more appropriate in many real‐world settings [21, 22].

Although Optilume demonstrates promising intermediate‐term outcomes (median follow‐up 29.5 months) with a minimally invasive profile, it currently represents a costlier option compared to standard endoscopic management, with the drug‐coated balloon priced at approximately 2650 Euros in Germany. Formal cost‐effectiveness analyses were beyond the scope of the present study. The outpatient setting may help reduce overall healthcare costs by avoiding prolonged hospitalization and inpatient resource utilization. At the same time, this may potentially reduce exposure to hospital‐associated pathogens.

Further studies are needed to better understand how prior interventions may influence treatment outcomes, which could aid in optimizing patient selection. For patients with bulbar urethral strictures who are either not suitable candidates for urethroplasty or prefer to avoid surgery, Optilume offers a valuable alternative that combines effective symptom relief with a less invasive approach.

Current EAU guidelines [17, 21] recommend Optilume for short bulbar strictures in patients who are not candidates for urethroplasty after recurrent treatments (EAU Guidelines 2025 LE 2a, SR B). However, our findings suggest that Optilume may offer more favorable outcomes when used earlier, potentially as a first‐line therapy in appropriately selected patients.

This study has several limitations. First, the relatively small sample size restricts the statistical power and limits the generalizability of the findings. However, considering that the use of drug‐coated balloon dilation in bulbar urethral strictures remains off‐label in many regions, the cohort reflects a real‐world clinical scenario characterized by careful patient selection and regulatory constraints. Second, the lack of a control group precludes direct comparisons with standard treatments such as internal urethrotomy or open urethroplasty. In our series, patients either declined urethroplasty or were not suitable surgical candidates, and therefore opted for treatment with Optilume. Third, the retrospective design introduces potential sources of bias, although the study was prompted by promising outcomes observed in initial cases managed with this approach. Finally, objective functional assessments (e.g., uroflowmetry or endoscopic follow‐up) were not routinely performed. Nonetheless, as highlighted before, therapeutic success in urethral stricture disease should increasingly focus on patient‐reported outcomes and the absence of retreatment. Although widely used in clinical practice, the IPSS is not a stricture‐specific patient‐reported outcome measure and may not fully capture anatomical treatment success.

Follow‐up was cross‐sectional, and assessments were performed at variable time points, reflecting routine clinical practice. As a result, follow‐up duration was not uniform across patients and may have differed between treatment‐naïve and previously treated subgroups. This may introduce bias when interpreting subgroup differences in symptomatic improvement. While functional outcomes are presented descriptively, subgroup‐specific mean ± SD values were not emphasized to avoid overinterpretation in a retrospective cohort with limited and imbalanced subgroup sizes.

## 5. Conclusions

Optilume demonstrated significant improvements in IPSS and QoL in patients with bulbar urethral strictures, with only one reintervention during a median follow‐up of 29.5 months, supporting favorable mid‐term outcomes with this minimally invasive treatment. Outcomes were similar between patients with and without previous interventions, although treatment‐naïve individuals tended to show slightly greater improvement, suggesting that earlier use of Optilume may merit further investigation.

Stricture length did not correlate with symptom improvement, indicating that within the evaluated range, clinical response appears independent of stricture length. By combining mechanical dilation with paclitaxel delivery, Optilume offers a valuable alternative for patients who are unsuitable for or decline urethroplasty.

From a healthcare‐system perspective, Optilume’s feasibility as an outpatient procedure may reduce resource utilization and recovery time, although device cost remains a relevant consideration.

Future studies should include larger prospective cohorts, incorporate standardized patient‐reported outcome measures, and compare Optilume with established treatment modalities to better define long‐term effectiveness, cost‐effectiveness, and optimal patient selection.

## Author Contributions

Juan Jose Menendez‐Suarez: protocol and project development, data collection and management, data analysis, manuscript writing, manuscript editing, and manuscript review.

Georgi Tosev: protocol and project development, data collection and management, data analysis, manuscript writing, manuscript editing, and manuscript review.

Hendrik Borgmann: protocol and project development, manuscript editing, and manuscript review.

Timur Kuru: protocol and project development, data collection and management, data analysis, manuscript writing, manuscript editing, and manuscript review.

Johannes Salem: protocol and project development, manuscript writing, manuscript editing, and manuscript review.

## Funding

No funds, grants, or other support were received. The authors have nothing to report. Open Access funding enabled and organized by Projekt DEAL.

## Disclosure

All authors have read and agreed to the published version of the manuscript. The funders had no role in the study design, data collection, or analysis, the decision to publish, or the preparation of the manuscript.

## Ethics Statement

Data​ acquisition and analysis were performed in compliance with protocols approved by the Landesärztekammer Baden−Württemberg (ethical approval number: F‐2023‐048, October 2023). Written informed consent was obtained from all participants prior to the study, and patients signed informed consent regarding publishing their data.

## Consent

Please see the Ethics Statement.

## Conflicts of Interest

The authors declare no conflicts of interest.

## Supporting Information

Supporting Table S1 summarizes the demographic and baseline clinical characteristics of the study cohort, including stricture features and pretreatment symptom scores. Supporting Figure S1 illustrates the relationship between stricture length and absolute change in IPSS (ΔIPSS) from baseline to follow‐up.

## Supporting information


**Supporting Information** Additional supporting information can be found online in the Supporting Information section.

## Data Availability

The data that support the findings of this study are available on request from the corresponding author. The data are not publicly available due to privacy or ethical restrictions.
