# Supplementary material for: Drug‐Coated Balloon (Optilume®) Shows a Low Reintervention Rate in Patients With Bulbar Urethral Strictures: Real‐World Data From Two German Centers
Source: Adv Urol. 2026 Mar 22;2026:8510976. doi: 10.1155/aiu/8510976 (PMC13137936; doi:10.1155/aiu/8510976)
Supplement: Supplementary file 1 — Supporting Information Additional supporting information can be found online in the Supporting Information section. [file AIU-2026-8510976-s001.docx]

| Variable | Mean ± SD | Median (Range) |
| --- | --- | --- |
| Age (years) | 59.6 ± 17.79 | 66.2 (24.3–90.2) |
| Number of Strictures | 1.1 ± 0.30 | 1.0 (1–2) |
| Number of Prior Treatments | 1.6 ± 2.29 | 1.0 (0–8) |
| Stricture Length (cm) | 1.8 ± 1.04 | 1.5 (0.5–4.0) |
| Follow up to Sept 2024 (months) | 27.6 ± 11.92 | 29.5 (9–44) |
| Pre-Treatment IPSS | 20.3 ± 8.08 | 19.5 (2–35) |
| Pre-Treatment QoL Score | 4.4 ± 1.19 | 5.0 (0–6) |

Supplementary Table S1 Demographic and Clinical History of the Study Cohort


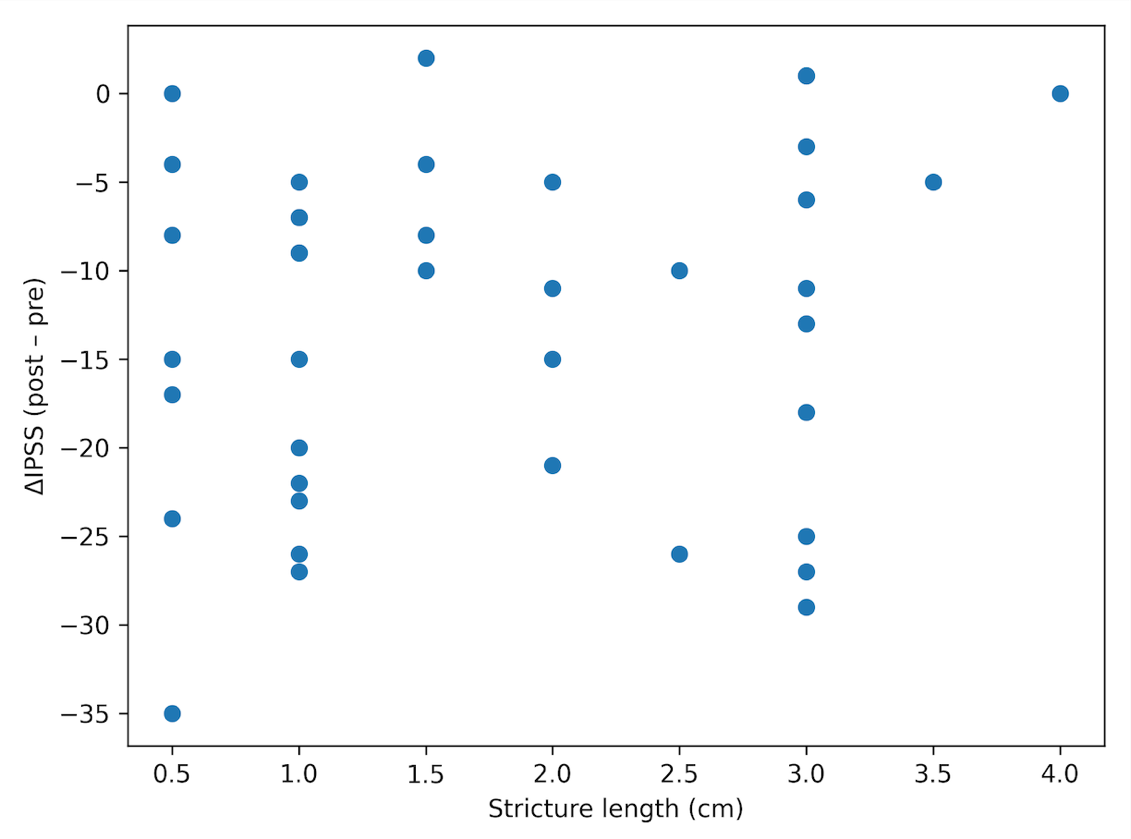


Supplementary Figure S1

Scatter plot showing the relationship between stricture length and absolute IPSS change (ΔIPSS) from baseline to follow-up. No clear association between stricture length and symptom improvement was observed.
